# Hints for the Sick-Room

**Published:** 1888-07-21

**Authors:** M. M. Basil

**Affiliations:** Author of "The Commoner Accidents to Life and Limb."


### HINTS FOR THE SICK-ROOM.

By M. M. Basil, M.A., M.B., C.M., Author of "The Commoner Accidents to Life and Limb."

### LEECHES, CUPPING, AND BLISTERS—*(continued)*.

The management of bleeding from leech-bites will be considered later on. It is very necessary to watch every case—especially if the patient is a child—that has been leeched, so that no continuous oozing of blood after the operation can be overlooked.

Wet cupping is a method of local bleeding. No one in this generation is likely to be asked to conduct the operation. It is mentioned here, to distinguish it from the next operation, known by an allied name. Dry cupping is a proceeding with the steps of which a nurse must make herself familiar. The operation aims essentially at forming a partial vacuum in a vessel of convenient size applied firmly at every point of contact against the skin. Cupping glasses of different sizes are used. A few drops of alcohol—methylated spirits, brandy, whisky, etc.—are poured into the cup, so as to moisten a large portion of its inner surface, the spirit is then lighted, allowed to burn nearly out, and the cup quickly and completely inverted against the body. Sometimes a piece of blotting-paper is soaked in alcohol, lighted, introduced into the cup, allowed to burn nearly out, and then the cup inverted. Accidental burns during dry cupping are nearly criminal. To avoid them just enough spirits must be used to freely moisten the cup; allow time merely sufficient to burn out the alcohol, but not long enough to cool the air in the cup; and last, the cup must be inverted quickly and completely, so as to exclude all fresh air from it. The smallest chink between any portion of the rim of the cup and the body will completely annul all precautions.

The cup must be held firmly against the skin until the part is felt to be drawn into it. It may then be left to itself for a quarter of an hour to half an hour or more. It is sometimes advisable to foment or poultice the part previous to dry cupping it; the operation is rendered more effectual by this means.

All that is necessary to remove the cupping-glass is to insinuate the nail gently between it and the skin, and press the body away from the rim of the glass. The slightest chink formed by this manœuvre will admit air into the glass and detach it at once. You must not pull away vigorously at the body of the cup; such an attempt causes the flesh to be drawn towards the cup all the more strongly, and bruises the skin against the hard rim.

Cupping-glasses may be applied carefully for extracting milk from the breasts, or for remedying retracted conditions of the nipples. In these circumstances the cup should not be heated much in the earlier attempts. A thick-lipped surgery bottle may also be used as breast extractors; but an ordinary soda-water bottle is not suitable, notwithstanding the statement to the contrary in many books.

Blisters are used frequently enough, and a large portion of their management is mainly in the hands of the nurse. The choice of the blistering agent will be determined for her by others. In the hearing of the patient it is often found convenient to put instructions on this point into technical language. It is therefore necessary to remember that the commonest of blistering agents—viz., the blistering fluid —is also known as *liquor epispasticus*. The advantage of remembering this name consists in its enabling the doctor to keep the patient in ignorance of the good things in store for him, and save him an anxiety of several hours' duration.

The seat and size of the blister will be determined in your instructions in each case. In absence of definite guidance, select such spots as are not exposed to pressure or contact with clothing. No blister should, as a rule, be larger than the area covered by the ear-piece of a stethoscope. In early attempts it would be advisable to ring the area with some olive oil or vaseline. This prevents the fluid running over parts which are not meant to be blistered. The area mapped out is painted with blistering fluid by means of a brush, and allowed to get dry before any clothing comes in contact with it. If it does not rise into a bleb or blister within eight to twelve hours, a linseed-meal poultice may be of advantage in raising it.

It is usual to puncture a blister or snip it with a pair of scissors at its lowest point. The fluid is best caught in a testtube or some cotton-wool. It should not be allowed to run over the healthy skin. One of the latest writers on nursing makes a statement which is not by any means in accordance with the practice followed by most medical men: "The aim, however, should always be to allow the fluid to become re-absorbed by leaving the blister undisturbed." Unless the same blister is to be kept open for a time by irritating ointments, it is best not to remove the covering which forms the surface of the blister. The fluid is let out, and the layer of skin which has been detached allowed to sink down like a flap, and protected with suitable dressings. Several blisters may thus be raised near each other at short intervals. These are known as flying blisters. They are raised in quick succession as soon as the previous one has healed. This form of continuous counter-irritation is less painful than an open blister.

The method in which a blister is managed after it risos makes all the difference between its being a veritable torture, or an application dreadful in name but comparatively painless in reality. The same remark holds good with regard to all forms of counter-irritation. It is not the application by itself which is so painful as the repeated torture which the badly-dressed surface causes before the parts are restored to their normal condition. The particular dressing to be used next to the blistered surface will be determined by the doctor, but it will be for the patient's friends or attendants to see that every margin of the blister is not merely covered but well overlapped by plenty of cotton-wool or other soft material. The dressings must be so well secured that no accident can possibly displace them.

Liquor epispasticus is the most convenient means of using Spanish flies for blistering, but in addition to the solution, a plaster made from the same material is also employed. The plaster, however, causes much more pain than the solution of cantharides, and becomes nearly unbearable if it is provided with an adhesive border all round. The reason for this difference is not very difficult to understand. Every counter-irritant produces local inflammation to a greater or less degree. One of the accompaniments of such inflammation is a tendency of the tissues to swell out or occupy more volume; in other words, there is an expansion of the tissues in all directions. This force of expansion is very considerable indeed, and quite sufficient to account for the agony which results when the part, especially the highly-sensitive skin, is bound down at every point to an inelastic material. Every point of contact becomes a telegraphic centre, from which the tender skin, in its attempt to wrench itself off from the plaster, sends up cries of distress to the brain.

In all cases where cantharides or Spanish flies are used for blistering, it is necessary to carefully watch for any alterations in the urine pointing to the presence of blood in it, pain in emptying the bladder, or frequent need to do so. Should the presence of any of these conditions be suspected, avoid applying cantharides to the patient again, and call the doctor's attention to the presence of the symptom. A similar caution holds good in cases where turpentine is applied to a large surface, but the risks here are comparatively small.

There are several other blistering agents in use besides Spanish flies. Strong liquid ammonia can be utilised by carefully painting the part with it, and covering it rapidly with an impermeable material; or a pad of blotting-paper dipped in the solution is placed over the body and quickly covered. A poultice must nearly always follow the application of ammonia. Avoid bringing the hand in contact with the fluid; eyes and nose will take good care of themselves in such cases. Before removing the stopper from a bottle containing ammonia, or any other strong chemical reagent, always direct the mouth of the bottle away from anyone in the room. The stopper must be held carefully, as a few drops of the reagent are sure to be found on it.

The ointment of the biniodide of mercury is a very efficient blister. Indeed, it is much more than a simple blister: it is a specific remedy in enlargements of the spleen from malarial fever (ague-cake), and in certain forms of gôitre. It is best used by placing the patient before a good fire with the part to be blistered exposed to the heat for some time. A piece of the ointment of the size of a large nutmeg is then gently rubbed in with a smooth paper-cutter or spatula. The fingers must not come in contact with the ointment. The patient is allowed to sit near the fire for some time, and then the blister is protected with plenty of cotton wool. This application cannot be repeated in a shorter time than a week to a fortnight.

The liniment of croton oil, the ointment of tartar emetic, and other preparations are used in very special cases. Some books recommend nurses to rub in these ointments quickly with their hands, and then wash them clean. It would be wise not to follow such advice. If an ointment is to counter-irritate the patient in any degree, it cannot but act on the nurse's fingers, and expose her to the risks of local or general poisoning from irritating discharges. A large painting brush will enable her to apply most of the liquid preparations, and a spatula or paper-cutter will answer in cases where ointments are used. A peculiar form of eczema, consisting of minute painful points on the volar surface of the ends of the fingers and under the free border of the nails, is noticed sometimes in nurses, doctors, and patients who use lotions to stimulate the growth of the hair. In every case a local irritant is at work, and may be traced by a searching investigation. Liniments or ointments may be rubbed in with the hand, provided they contain no agents that stain the hand, or act locally on the skin during a short time.

(*To be continued.*)

